# The Occurrence of Biogenic Amines and Determination of Biogenic Amine-Producing Lactic Acid Bacteria in *Kkakdugi* and *Chonggak* Kimchi

**DOI:** 10.3390/foods8020073

**Published:** 2019-02-14

**Authors:** Young Hun Jin, Jae Hoan Lee, Young Kyung Park, Jun-Hee Lee, Jae-Hyung Mah

**Affiliations:** Department of Food and Biotechnology, Korea University, 2511 Sejong-ro, Sejong 30019, Korea; younghoonjin3090@korea.ac.kr (Y.H.J.); jae-lee@korea.ac.kr (J.H.L.); eskimo@korea.ac.kr (Y.K.P.); bory92@korea.ac.kr (J.-H.L.)

**Keywords:** kimchi, *Kkakdugi*, *Chonggak* kimchi, radish kimchi, biogenic amines, tyramine, lactic acid bacteria, *Lactobacillus brevis*

## Abstract

In this study, biogenic amine content in two types of fermented radish kimchi (*Kkakdugi* and *Chonggak* kimchi) was determined by high performance liquid chromatography (HPLC). While most samples had low levels of biogenic amines, some samples contained histamine content over the toxicity limit. Additionally, significant amounts of total biogenic amines were detected in certain samples due to high levels of putrefactive amines. As one of the significant factors influencing biogenic amine content in both radish kimchi, *Myeolchi-aekjoet* appeared to be important source of histamine. Besides, tyramine-producing strains of lactic acid bacteria existed in both radish kimchi. Through 16s rRNA sequencing analysis, the dominant species of tyramine-producing strains was identified as *Lactobacillus brevis*, which suggests that the species is responsible for tyramine formation in both radish kimchi. During fermentation, a higher tyramine accumulation was observed in both radish kimchi when *L. brevis* strains were used as inocula. The addition of *Myeolchi-aekjeot* affected the initial concentrations of histamine and cadaverine in both radish kimchi. Therefore, this study suggests that reducing the ratio of *Myeolchi-aekjeot* to other ingredients (and/or using *Myeolchi-aekjeot* with low biogenic amine content) and using starter cultures with ability to degrade and/or inability to produce biogenic amines would be effective in reducing biogenic amine content in *Kkakdugi* and *Chonggak* kimchi.

## 1. Introduction

Biogenic amines (BA) have been considered to be toxic compounds in foods. Several authors have proposed the maximum tolerable limits of some toxicologically important BA in foods as follows: histamine, 100 mg/kg; tyramine, 100–800 mg/kg; β-phenylethylamine, 30 mg/kg; total BA, 1000 mg/kg [1,2]. In addition, polyamines such as putrescine and cadaverine have been known to potentiate the toxicity of BA, especially histamine and tyramine, in foods, although they are less toxic [1]. Consumption of foods containing excessive BA may cause symptoms such as migraines, sweating, nausea, hypotension, and hypertension, unless human intestinal amine oxidases—such as monoamine oxidase (MAO), diamine oxidase (DAO), and polyamine oxidase (PAO)—quickly metabolize and detoxify BA [3]. Thus, it is important to know that, although relatively low levels of BA naturally exist in common foods, microbial decarboxylation of amino acids may sometimes lead to a significant increment of BA in fermented or contaminated foods [2]. In lactic acid fermented foods such as cheese and fermented sausage, some species of lactic acid bacteria (LAB) have been considered as producers of BA, particularly tyramine [4]. On the other hand, several reports have indicated that use of LAB starter cultures unable to produce BA may reduce BA accumulation during fermentation and storage [5,6].

Kimchi is a generic term of Korean traditional lactic fermented vegetables. According to Codex standard [7], for preparation of kimchi, salted Chinese cabbage (as a main ingredient) is mixed with seasoning paste consisting of red pepper powder, radish, garlic, green onion, and ginger, and then fermented properly, however, which, in reality, refers to *Baechu* kimchi. Alongside the Chinese cabbage, various vegetables such as radish, ponytail radish, cucumber, and green onion are also used as main ingredients of kimchi depending on kimchi varieties in Korea [8]. Among numerous kimchi varieties prepared with different vegetables, *Baechu* kimchi, *Kkakdugi* (diced radish kimchi), and *Chonggak* kimchi (ponytail radish kimchi) are the most popular varieties of kimchi in Korea [9]. In the meantime, for improving sensory quality of kimchi, various types of salted and fermented seafood (*Jeotgal*) and sauces thereof (*Aekjeot*) are usually used for kimchi preparation in Korea [10]. Particularly, *Myeolchi-jeotgal* (salted and fermented anchovy), *Saeu-jeotgal* (salted and fermented shrimp), *Myeolchi-aekjeot* (a sauce prepared from *Myeolchi-jeotgal*) are commonly used *Jeotgal* and *Aekjeot* [11]. As *Jeotgal* and *Aekjeot* contain high levels of proteins and amino acids, when kimchi is prepared with them, BA accumulation may occur during kimchi fermentation [12]. Hence, several authors have intensively investigated BA content and BA-producing LAB in *Baechu* kimchi [13,14,15]. On the other hand, there is a lack of study on BA content and BA-producing LAB in *Kkakdugi* and *Chonggak* kimchi, although the two types of radish kimchi are as popular as *Baechu* kimchi in Korea.

In this study, therefore, BA content in *Kkakdugi* and *Chonggak* kimchi was determined to evaluate BA-related risks. Several possible contributing factors to BA content, including physicochemical properties and microbial BA production, were also investigated in the study. Finally, fermentation of both radish kimchi was carried out to determine the most important bacterial species contributing to BA formation in the radish kimchi, employing LAB strains with distinguishable BA-producing activities as fermenting microorganisms. This is the first study describing that *Lactobacillus brevis* is the species responsible for tyramine formation in kimchi variety throughout fermentation period.

## 2. Materials and Methods

### 2.1. Sampling

Two types of radish kimchi (*Kkakdugi* and *Chonggak* kimchi) samples of five popular kimchi manufacturers made within 30 days were obtained from the retail markets. After arrival, samples were stored at 4 °C or immediately analyzed for BA content, physicochemical parameters, and microbial measurement.

### 2.2. Physicochemical Measurements

pH, acidity, salinity, and water activity of *Kkakdugi* and *Chonggak* kimchi samples were determined. The pH of the samples was determined by Orion 3-star Benchtop pH meter (Thermo Scientific, Waltham, MA, USA). Acidity and salinity were measured according to the AOAC method [16]. The water activity was determined by water activity meter (AquaLab Pre; Meter Group, Inc., Pullman, WA, USA).

### 2.3. Microbial Measurement, Isolation, and Identification of Strains

Lactic acid bacterial counts and total aerobic bacterial counts were determined on de Man, Rogosa, and Sharpe (MRS, Laboratorios Conda Co., Madrid, Spain) agar and Plate Count Agar (PCA, Difco, Becton Dickinson, Sparks, MD, USA). According to manufacturer’s instructions, MRS agar was incubated at 37 °C for 48–72 h, and PCA at 37 °C for 24 h. After incubation, enumeration was carried out on plates with 30–300 colonies.

LAB strains were isolated on MRS agar. Individual colonies on MRS agar were randomly selected and streaked on the same media. The single colonies were transferred to MRS broth at 37 °C for 48–72 h. Then, the cultured broth was stored in the presence of 20% glycerol (*v*/*v*) at −80 °C. In *Kkakdugi* and *Chonggak* kimchi samples, 130 and 120 LAB strains were isolated, respectively. The strains were identified by 16s rRNA gene sequence analysis with the universal bacterial primer pair (518F and 805R, Solgent Co., Daejeon, Korea).

### 2.4. BA Extraction from Samples and Bacterial Cultures for HPLC Analysis

BA extraction from *Kkakdugi* and *Chonggak* kimchi samples was conducted by the methods developed by Eerola et al. [17], with minor modification. The sample broth (5 g) was mixed with 20 mL of perchloric acid (0.4 M). The mixture was incubated at 4 °C for 2 h and centrifuged at 3000× *g* at 4 °C for 10 min. After collecting the supernatant, the pellet was extracted again with equal volumes of perchloric acid under the same conditions. The total volume of supernatant was adjusted to 50 mL with perchloric acid. The extract was filtered using Whatman paper no. 1 and stored before analysis.

BA extraction from bacterial cultures was carried out based on the procedures described by Ben-Gigirey et al. [18,19], with minor modification. A loopful of a strain was inoculated in 5 mL of BA production assay medium. The compositions of BA production assay medium are as follows: MRS broth with 0.5% of L-ornithine monohydrochloride, L-lysine monohydrochloride, L-histidine monohydrochloride monohydrate, and L-tyrosine disodium salt hydrate (all Sigma-Aldrich Chemical Co., St. Louis, MO, USA); 0.0005% of pyridoxal-HCl (Sigma-Aldrich); pH of the broth was adjusted to 5.8 by adding hydrochloride solution (2 M). After incubating the strain at 37 °C for 48 h, 100 μL of the culture was inoculated into the same broth and incubated under the same conditions. Subsequently, after being mixed with 0.4 M perchloric acid at a volume ratio of 1:9, the mixture was incubated at 4 °C for 2 h and stored before analysis.

### 2.5. Preparation of Standard Solutions for HPLC Analysis

Tryptamine, β-phenylethylamine hydrochloride, putrescine dihydrochloride, cadaverine dihydrochloride, histamine dihydrochloride, tyramine hydrochloride, spermidine trihydrochloride, and spermine tetrahydrochloride (all Sigma-Aldrich) were used for standard solutions, and 1,7-diaminoheptane (Sigma-Aldrich) was applied for an internal standard. The concentrations of all standard solutions were adjusted to 0, 10, 50, 100, and 1000 ppm.

### 2.6. Derivatization of Extracts and Standards

The procedures of derivatization of BA in the extract were carried out by the method developed by Eerola et al. [17]. Briefly, 200 μL of 2 M sodium hydroxide and 300 μL of saturated sodium bicarbonate were added to 1 mL of the extract/standard solutions. Then, 2 mL of 1% dansyl chloride solution (dissolved in acetone) was mixed with the solution and then incubated for 45 min at 40 °C in dark room. The incubated solution was mixed with 100 μL of 25% ammonium hydroxide and reacted for 30 min at room temperature. The volume of the sample solution was adjusted to 5 mL by adding acetonitrile. The sample solution was centrifuged at 3000× *g* for 5 min, and the supernatant was filtered by using a 0.2 μm-pore-size filter (Millipore Co., Bedford, MA, USA).

### 2.7. HPLC Analysis

HPLC analysis was carried out according to the procedure developed by Eerola et al. [17] and modified by Ben-Gigirey et al. [18]. YL9100 HPLC system equipped with YL9120 UV–vis detector (all Younglin, Anyang, Korea) was employed and the data were analyzed with Autochro-3000 data system (Younglin). For the gradient HPLC method, 0.1 M ammonium acetate (solvent A; Sigma-Aldrich) and HPLC-grade acetonitrile (solvent B; SK chemicals, Ulsan, Korea) were used as the mobile phases. The chromatographic separation was carried out using Nova-Pak C18 column (4 μm, 4.6 × 150 mm; Waters, Milford, MA, USA) held in 40 °C at a flow rate of 1 mL/min. The gradient elution mode was as follows; 50:50 (A:B) to 10:90 for 19 min, 50:50 at 20 min, isocratic with 50:50 before next analysis. The analysis was conducted at 254 nm, and 10 μL of the sample solution was injected.

The detection limits were within the range of 0.01 to 0.10 mg/kg for food matrices [20]. The validation parameters, including detection limits, of the analytical procedure used in the study were reported in our earlier study [20]. Figure S1 illustrates the procedure, from extraction to HPLC analysis, for BA analysis.

### 2.8. Fermentation of Two Types of Radish Kimchi: *Kkakdugi* and *Chonggak* Kimchi

For preparation of *Kkakdugi* and *Chonggak* kimchi, diced white radish (2 × 2 × 2 cm^3^) or halved ponytail radish were soaked in 10% w/v salt brine for 30 min, respectively. Then, each salted radish was rinsed with tap water three times and drained for 3 h. *Kkakdugi* and *Chonggak* kimchi samples were prepared in triplicate, as shown in Table 1, according to the standard recipes developed by the National Institute of Agricultural Sciences [21]. The salinity of all samples was adjusted to 2.5%. The *Kkakdugi* and *Chonggak* kimchi samples were divided into five experimental groups, respectively, based on the presence or absence of *Myeolchi-aekjeot* and *Saeu-jeotgal* and LAB inoculum. The experimental groups designed for the present study were B group (“Blank” samples prepared with neither *Myeolchi-aekjeot* and *Saeu-jeotgal* nor inoculum), C group (“Control” samples prepared with *Myeolchi-aekjeot* and *Saeu-jeotgal*, but without inoculum), PC group (“Positive Control” samples prepared with *Myeolchi-aekjeot* and *Saeu-jeotgal*, and inoculated with *L. brevis* JCM 1170 as a reference strain), LB group (“L. brevis” samples prepared with *Myeolchi-aekjeot* and *Saeu-jeotgal*, and inoculated with tyramine-producing *L. brevis* strains, i.e., KD3M5 strain for *Kkakdugi* and CG2M15 strain for *Chonggak* kimchi, respectively), and LP group (“L. plantarum” samples prepared with *Myeolchi-aekjeot* and *Saeu-jeotgal*, and inoculated with *L. plantarum* strains, i.e., KD3M15 strain for *Kkakdugi* and CG3M21 strain for *Chonggak* kimchi, respectively). The samples belonging to respective experimental groups were fermented at 25 °C for three days. Changes on the physicochemical and microbial properties, and BA content were measured in triplicate during fermentation.

### 2.9. Statistical Analyses

Statistical analyses were performed with Minitab statistical software version 12.11 (Minitab Inc. State College, PA, USA). The data were presented as means ± standard deviations of the three independent replicates. The mean values were compared by one-way analysis of variance (ANOVA) with Tukey’s honest significant difference (HSD) test and a probability (*p*) values of less than 0.05 were considered statistically significant.

## 3. Results and Discussion

### 3.1. Determination of BA Content in Radish Kimchi: Kkakdugi and Chonggak Kimchi

As shown in Table 2, BA content in *Kkakdugi* and *Chonggak* kimchi samples produced by popular manufacturers in Korea was determined, and human health risk of BA in both radish kimchi was estimated based on the suggestions of both Ten Brink et al. [1] and Silla Santos [2]. In all the samples of *Kkakdugi* and *Chonggak* kimchi, low levels of tyramine (<100 mg/kg), tryptamine, β-phenylethylamine, spermidine, and spermine (<30 mg/kg) were detected, which are within safe levels for human consumption. However, one *Kkakdugi* sample (KD2) had 127.78 ± 26.78 mg/kg of histamine, which is over the toxicity limit (100 mg/kg) suggested by Ten Brink et al. [1]. Another *Kkakdugi* sample (KD5) contained putrescine and cadaverine at concentrations of 982.32 ± 19.42 mg/kg and 124.60 ± 108.78 mg/kg, respectively, consequently exceeding the 1000 mg/kg limit for total BA which is considered to provoke toxicity [2]. In *Chonggak* kimchi samples, 131.20 ± 7.90 mg/kg of histamine was detected in one sample (CG5), which also contained 853.7 ± 36.80 mg/kg of putrescine and 112.10 ± 3.60 mg/kg of cadaverine. The amounts of histamine and total BA in the sample were found to exceed toxicity limits. Meanwhile, the BA content detected in both types of radish kimchi samples varied widely in the present study, which is similar to respective BA levels in *Baechu* kimchi reported previously [13,22]. On the other hand, Mah et al. [12] reported lower concentrations of putrescine, cadaverine, histamine, tyramine, spermidine, and spermine in both *Kkakdugi* and *Chonggak* kimchi than those detected in the same kinds of kimchi used in this study. This may be due to the differences in manufacturing methods, main ingredients, and storage conditions between kimchi samples used in the present and previous studies [9]. In the meantime, Mah et al. [12] also reported that the amounts of tyramine and other BA increased during the ripening of *Baechu* kimchi. Therefore, although tyramine was detected at low levels in all the samples of *Kkakdugi* and *Chonggak* kimchi in the present study, the significance and risk of tyramine formation in both types of radish kimchi should not be overlooked.

According to Tsai et al. [13], a high level of histamine in kimchi may result from the addition of salted and fermented fish products. *Myeolchi-aekjeot* is the most widely used salted and fermented fish product for the preparation of kimchi variety, and approximately 2–4% of *Kkakdugi* (on the basis of weight percent) and 2–5% of *Chonggak* kimchi, respectively, are commonly added to main ingredients during kimchi preparation [21,23,24,25,26]. *Saeu-jeotgal* is also added, alone or together with *Myeolchi-aekjeot*, to main ingredients of kimchi, but Mah et al. [12] reported that *Myeolchi-aekjeot* contains a significantly higher level of histamine (up to 1154.7 mg/kg) than *Saeu-jeotgal*. In this study, all radish kimchi samples were prepared with both *Myeolchi-aekjeot* and *Saeu-jeotgal* as ingredients. Altogether, the excessive level of histamine in several radish kimchi samples could be due to the amount of added *Myeolchi-aekjeot* with high histamine content. Unfortunately, the food labels of the samples used in this study just provided the list of ingredients.

An overdose of histamine may provoke undesirable symptoms such as a migraine, sweating, and hypotension [3]. In addition, high levels of putrescine and cadaverine can potentiate histamine toxicity by inhibiting intestinal diamine oxidase and histamine-N-methyltransferase [27] and potentially react with nitrites to form carcinogenic N-nitrosamines [28]. Taking this into account, although most *Kkakdugi* and *Chonggak* kimchi samples seem to be safe for consumption, the fact that several samples contained relatively high levels of putrescine and cadaverine in the present study indicates that it is necessary to monitor and reduce BA content, particularly histamine, putrescine, and cadaverine.

### 3.2. Physicochemical and Microbial Properties of Radish Kimchi: Kkakdugi and Chonggak Kimchi

To predict possible reasons as to why some samples of two types of radish kimchi contained higher levels of BA, pH, acidity, salinity, water activity (a_w_), and lactic acid bacterial and total aerobic bacterial counts of *Kkakdugi* and *Chonggak* kimchi samples were determined. In *Kkakdugi* samples, the values of the parameters were as follows: pH, 4.16 ± 0.17 (minimum to maximum range of 3.94–4.41); acidity (%), 0.86 ± 0.31 (0.51–1.27); salinity (%), 3.36 ± 1.21 (1.40–4.50); a_w_, 0.983 ± 0.003 (0.977–0.988); lactic acid bacterial counts, 8.52 ± 0.61 Log CFU/mL (7.88–9.38 Log CFU/mL); total aerobic bacterial counts, 8.37 ± 0.96 Log CFU/mL (6.83–9.32 Log CFU/mL). In case of *Chonggak* kimchi samples, the measured values were as follows: pH, 4.96 ± 1.17 (3.98–6.36); acidity (%), 0.71 ± 0.43 (0.19–1.10); salinity (%), 3.83 ± 1.67 (2.15–6.48); a_w_, 0.984 ± 0.004 (0.979–0.991); lactic acid bacterial counts, 7.83 ± 0.48 Log CFU/mL (7.42–8.60 Log CFU/mL); total aerobic bacterial counts, 8.18 ± 1.07 Log CFU/mL (6.88–9.48 Log CFU/mL). The values are in accordance with those of previous reports [13,29]. Linear regression analysis was performed to determine the contributors influencing BA content. Results revealed weak correlations between physiochemical parameters, as well as microbial properties, and BA content (data not shown). Nonetheless, several reports have shown that physicochemical and microbial properties may affect BA content in fermented foods [2,30,31]. Altogether, the results indicate that, besides physicochemical and microbial properties, there are complex factors affecting BA content in both radish kimchi, for instance, kinds of salted and fermented fish products used for kimchi preparation as described above.

### 3.3. BA Production by LAB Strains Isolated from Radish Kimchi: Kkakdugi and Chonggak Kimchi

BA production by LAB strains isolated from *Kkakdugi* and *Chonggak* kimchi samples was examined to determine BA-producing LAB species in two types of radish kimchi. All the strains showed low production (below the detection limit) of tryptamine, β-phenylethylamine, putrescine, cadaverine, histamine, spermidine, and spermine. However, 39 strains (30%) of 130 LAB isolated from *Kkakdugi* samples produced higher levels of tyramine (287.23–386.17 μg/mL) than other strains (below the detection limit). Among the 120 LAB strains isolated from *Chonggak* kimchi, 16 strains (13%) also showed a stronger tyramine production capability (260.93–339.56 μg/mL), while other strains revealed lower capability (below the detection limit). In addition, the tyramine-producing LAB strains, which were isolated from either *Kkakdugi* or *Chonggak* kimchi samples, revealed a similar ability to produce tyramine, as described right above. Meanwhile, despite the low level of tyramine detected in all the samples of *Kkakdugi* and *Chonggak* kimchi, the fact that parts of LAB strains isolated from both radish kimchi samples were highly capable of producing tyramine supports that tyramine increment may occur during the ripening of the kimchi [12].

To further determine microorganisms responsible for BA formation in radish kimchi at species level, the strains were divided into two groups: (i) 55 tyramine-producing LAB strains (39 strains from *Kkakdugi*; 16 strains from *Chonggak* kimchi) and (ii) 195 LAB strains unable to produce BA. In the two groups, several strains were randomly selected and subsequently identified based on 16s rRNA sequencing analysis. Then, the selected strains able to produce tyramine were all identified as *L. brevis*, which indicates that the species is probably responsible for tyramine formation in both types of radish kimchi. On the other hand, the selected strains unable to produce BA were identified as *Leuconostoc* (*Leu.*) *mesenteroides*, *Weissella cibaria*, *W*. *paramesenteroides, L. pentosus,* and *L. plantarum*. The results are in agreement with previous reports in which *Leuconostoc*, *Weissella*, and *Lactobacillus* spp. were suggested to be responsible for kimchi fermentation [8,32]. Meanwhile, tyramine production by *L. brevis* in various fermented foods, including wine and fermented sausage, as well as *Baechu* kimchi, has been previously reported [14,33,34]. In the reports, tyramine production by *L. brevis* isolated from wine ranged from 441.6 to 1070.0 μg/mL, which is higher than that of the present study. On the contrary, *L. brevis* isolated from fermented sausage and *Baechu* kimchi produced tyramine at the range from 138.51 to 169.47 μg/mL and from 282 to 388 μg/mL, respectively, which are similar or lower than that of this study. In addition, several authors also isolated tyramine-producing *Leu. mesenteroides*, *W. cibaria*, and *W*. *paramesenteroides* from *Baechu* kimchi [14,15] and *L. plantarum* from wine [35]. Interestingly, as described right above, there are somewhat disparate results between the present and previous studies, which indicates that the strains belonging to the same species may possess different ability to produce tyramine especially depending upon the kinds of foods. Thus, microbial BA production in radish kimchi is likely determined at strain level, probably adapting to the respective food ecosystems, as suggested by previous reports [36,37]. Another implication is that the strains unable to produce BA isolated in the current study have potential as starter cultures for kimchi fermentation. Further investigations are needed to use them as starter cultures, which may involve tests to examine if the strains fulfill the criteria of starter culture, including the technical properties of strains, food safety requirements, and quality expectations [38].

### 3.4. Changes in Tyramine and Other BA Content during Fermentation of Radish Kimchi: Kkakdugi and Chonggak Kimchi

Fermentation of *Kkakdugi* and *Chonggak* kimchi was performed to investigate the influences of *Myeolchi-aekjeot* (together with *Saeu-jeotgal*) and LAB strains (particularly *L. brevis*) on BA content (especially tyramine) of both radish kimchi. Five groups of *Kkakdugi* and *Chonggak* kimchi samples were prepared based on the presence or absence of *Myeolchi-aekjeot* and types of LAB inocula. *L. brevis* strains of KD3M5 and CG2M15 with the highest tyramine production activity among the identified tyramine-producing LAB strains were used to see if the species is practically responsible for tyramine formation during fermentation of *Kkakdugi* and *Chonggak* kimchi. On the other hand, *L. plantarum* strains of KD3M15 and CG3M21 unable to produce BA were used for two reasons. (i) *L. plantarum*, like *L. brevis*, is predominant species in kimchi [39]. (ii) Differently from *L. brevis*, *L. plantarum* has been found to be negative for tyramine production in the present and previous studies [33,40,41].

**Figure 1:**
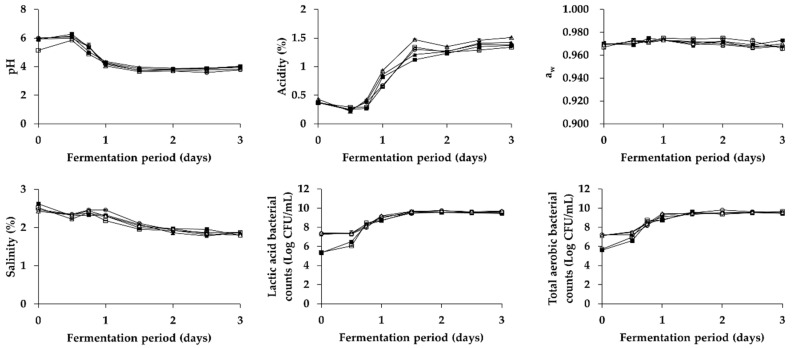
Changes in physicochemical and microbial properties of *Kkakdugi* during fermentation. □: B (no addition of *Myeolchi-aekjeot* and *Saeu-jeotgal*, no inoculum), ■: C (addition of *Myeolchi-aekjeot* and *Saeu-jeotgal*, no inoculum), ▲: PC (addition of *Myeolchi-aekjeot* and *Saeu-jeotgal*, *L. brevis* JCM 1170), △: LB (addition of *Myeolchi-aekjeot* and *Saeu-jeotgal*, *L. brevis* KD3M5), ⚬: LP (addition of *Myeolchi-aekjeot* and *Saeu-jeotgal*, *L. plantarum* KD3M15).

**Figure 2:**
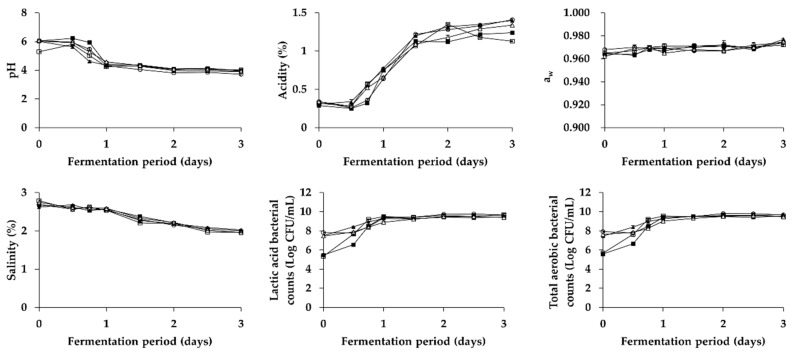
Changes in physicochemical and microbial properties of *Chonggak* kimchi during fermentation. □: B (no addition of *Myeolchi-aekjeot* and *Saeu-jeotgal*, no inoculum), ■: C (addition of *Myeolchi-aekjeot* and *Saeu-jeotgal*, no inoculum), ▲: PC (addition of *Myeolchi-aekjeot* and *Saeu-jeotgal*, *L. brevis* JCM 1170), △: LB (addition of *Myeolchi-aekjeot* and *Saeu-jeotgal*, *L. brevis* CG2M15), ⚬: LP (addition of *Myeolchi-aekjeot* and *Saeu-jeotgal*, *L. plantarum* CG3M21).

As shown in Figure 1 and Figure 2, changes in physicochemical and microbial properties of *Kkakdugi* and *Chonggak* kimchi during the fermentation for 3 days were similar with those of several previous reports [25,29,42]. In detail, the pH of all radish kimchi groups decreased during day 1 of fermentation, and stayed constantly thereafter. On the contrary, the counts of total aerobic bacteria and lactic acid bacteria, and the acidity of all radish kimchi groups increased during day 1 and day 2, respectively, and remained constantly thereafter, which indicates that an appropriate fermentation process of *Kkakdugi* and *Chonggak* kimchi took place. It is mention worthy that the initial pH of C, PC, LB, and LP groups of both radish kimchi was slightly higher than that of B group, which might be because the neutral pH of *Saeu-jeotgal* affected the pH values of the former groups [43]. Nonetheless, the initial acidity of all groups, belonging to either *Kkakdugi* or *Chonggak* kimchi, was similar to each other. The salinity of all radish kimchi groups decreased slightly during fermentation. According to Shin, Ann, and Kim [44], osmosis between radish and broth (containing seasoning paste) occurs during fermentation, which results in a steady reduction of salinity. Regardless of the drop in salinity, water activity of all radish kimchi groups was constant during fermentation. In addition, the initial counts of total aerobic bacteria and lactic acid bacteria of PC, LB, and LP groups inoculated with any of LAB strains were higher than those of B and C groups to be fermented naturally without any inocula, as expected.

**Figure 3:**
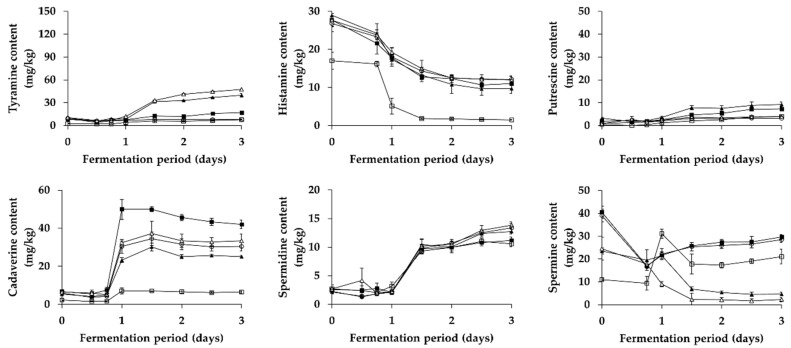
Changes in BA content in *Kkakdugi* during fermentation. □: B (no addition of *Myeolchi-aekjeot* and *Saeu-jeotgal*, no inoculum), ■: C (addition of *Myeolchi-aekjeot* and *Saeu-jeotgal*, no inoculum), ▲: PC (addition of *Myeolchi-aekjeot* and *Saeu-jeotgal*, *L. brevis* JCM 1170), △: LB (addition of *Myeolchi-aekjeot* and *Saeu-jeotgal*, *L. brevis* KD3M5), ⚬: LP (addition of *Myeolchi-aekjeot* and *Saeu-jeotgal*, *L. plantarum* KD3M15).

**Figure 4:**
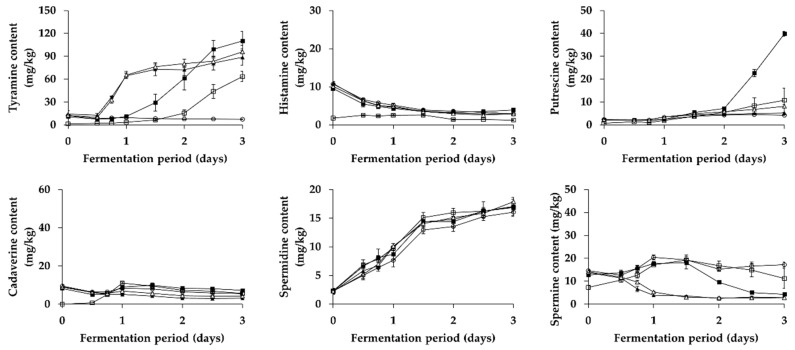
Changes in BA content in *Chonggak* kimchi during fermentation. □: B (no addition of *Myeolchi-aekjeot* and *Saeu-jeotgal*, no inoculum), ■: C (addition of *Myeolchi-aekjeot* and *Saeu-jeotgal*, no inoculum), ▲: PC (addition of *Myeolchi-aekjeot* and *Saeu-jeotgal*, *L. brevis* JCM 1170), △: LB (addition of *Myeolchi-aekjeot* and *Saeu-jeotgal*, *L. brevis* CG2M15), ⚬: LP (addition of *Myeolchi-aekjeot* and *Saeu-jeotgal*, *L. plantarum* CG3M21).

Changes in BA content (except for tryptamine and β-phenylethylamine not detected) during fermentation of *Kkakdugi* and *Chonggak* kimchi were shown in Figure 3 and Figure 4, respectively. There appeared an increment of tyramine content in most groups (except for LP group) of both radish kimchi over the fermentation period, probably resulting from tyramine production by either inoculated or indigenous *L. brevis* strains (refer to Section 3.3). Also, the increment of tyramine content in PC and LB groups was higher than that in B and C groups of both radish kimchi (except for day 3 of *Chonggak* kimchi fermentation). This might be due to higher lactic acid bacterial counts of PC and LB groups, resulting from the inoculation of tyramine-producing *L. brevis* strains, than those of B and C groups of both radish kimchi. In the meantime, tyramine content in B and C groups of *Chonggak* kimchi steadily increased during fermentation, while that in the same groups of *Kkakdugi* increased slightly (but at a low level compared to *Chonggak* kimchi), both of which are likely associated with tyramine production by indigenous LAB strains (probably *L. brevis*). The observations are consistent with previous reports described right below. In short, Choi et al. [45] reported a dramatic increase of tyramine during natural fermentation of *Baechu* kimchi, whereas Kim et al. [46] reported that *Baechu* kimchi had a constantly low level of tyramine during natural fermentation. It is also noteworthy that, in the case of *Chonggak* kimchi, tyramine content in PC and LB groups dramatically increased during day 1 of fermentation, which was higher (and also showed a faster increment) than that in the same groups of *Kkakdugi*. The results, together with the comparison of tyramine content in B and C groups between two types of radish kimchi described above, can be explained by two speculations. The first is the difference in the ability of *L. brevis* strains to produce tyramine. The second is the distinguishable adaptation of the strains to different food ecosystems, i.e., differences in the main ingredients and/or ratio of ingredients in seasoning paste between two types of radish kimchi. Since KD3M5 strain served as an inoculum for *Kkakdugi* revealed a stronger ability to produce tyramine (377.35 ± 4.36 μg/mL) than CG2M15 strain for *Chonggak* kimchi (328.48 ± 2.61 μg/mL) when compared in vitro (refer to Section 3.3), the second speculation seems to be more probable than the first one. In addition, it is well known that bacteria produce BA to neutralize acidic environments as part of homeostatic regulation [47]. In this study, however, both radish kimchi samples of PC and LB groups showed similar patterns of acidity changes, so that the homeostatic regulation was excluded from possible reasons. Either way, there seem to be much complicated cross effects by the combinations of factors influencing the intensity of BA production by LAB during fermentation of kimchi variety. Interestingly, LP group of both radish kimchi had significantly lower levels of tyramine than the other groups. Thus, it seems that *L. plantarum* strains unable to produce BA in vitro not only have incapability of producing BA during fermentation, but also may inhibit tyramine production by indigenous LAB strains. This indicates the applicability of this species as a starter culture for reducing BA in kimchi variety.

Differently from tyramine, histamine content in all groups of both radish kimchi gradually decreased during fermentation. This result might be because there were some indigenous LAB strains with histamine-degrading activity. Similarly, Kim et al. [48] reported a significant reduction of histamine content in *Baechu* kimchi inoculated with type strains of different LAB species including *L. sakei*, *L. plantarum*, *Leu. carnosum*, and *Leu. mesenteroides*, when compared with non-inoculated kimchi, suggesting that some LAB stains in kimchi are capable of degrading histamine. Meanwhile, the experimental groups of *Kkakdugi* and *Chonggak* kimchi prepared with *Myeolchi-aekjeot* (C, PC, LB, and LP groups) contained a significantly higher level of histamine than B group, which is in accordance with the suggestion of previous studies [12,22]. In the studies, the authors assumed that histamine level in *Baechu* kimchi could be affected by histamine in *Myeolchi-aekjeot*. Taking this into account, histamine content of *Kkakdugi* and *Chonggak* kimchi in the present study seems to come from *Myeolchi-aekjeot* rather than microbial histamine production during fermentation.

Putrescine and spermidine content steadily increased in all groups of *Kkakdugi* and *Chonggak* kimchi during fermentation, which is in agreement with previous reports [12,46]. There was a small and insignificant difference in putrescine and spermidine content among the groups of both radish kimchi during fermentation, which indicates that LAB strains—including *L. brevis* and *L. plantarum*—produced the polyamines during fermentation. Meanwhile, the initial concentrations putrescine and spermidine in *Kkakdugi* and *Chonggak* kimchi might be come from main ingredients, i.e., white radish and ponytail radish, respectively. In addition, a sharp increment of putrescine was observed during day 3 of fermentation, in the case of C group of *Chonggak* kimchi. To ignore the possibility of outliers, the fermentation experiment was repeatedly performed; however, the same results were observed, and the reason for such observation was not clear.

Somewhat differently from above, cadaverine content in all groups of *Kkakdugi* and *Chonggak* kimchi showed an increment during day 1 of fermentation and slight decline thereafter, although the increased cadaverine amount was mostly higher in *Kkakdugi* than in *Chonggak* kimchi. The difference in the intensity of cadaverine formation between two types of radish kimchi seems to be attributed to the complex combinations of factors described above to explain difference in the kinetics of tyramine formation between two radish kimchi. Interestingly, the initial cadaverine content in C, PC, LB, and LP groups of both radish kimchi was higher than that in B group, which might be come from *Myeolchi-aekjeot* rather than *Saeu-jeotgal*. The speculation is supported by a study by Cho et al. [22] who reported a significantly higher level of cadaverine in *Myeolchi-aekjeot* (up to 263.6 mg/kg) than that in *Saeu-jeotgal* (up to 7.0 mg/kg). For both radish kimchi, C group contained the highest level of cadaverine, as compared to the other groups, over the fermentation period. This may be explained by a presumption that while cadaverine-producing bacteria derived from *Myeolchi-aekjeot* are probably responsible for cadaverine formation during fermentation of both radish kimchi, LAB strains (*L. brevis* and *L. plantarum*) served as inocula are probably capable of degrading cadaverine. Supporting this presumption, Mah et al. [49] reported that *Bacillus* strains isolated from *Myeolchi-jeotgal* were highly capable of producing cadaverine. Capozzi et al. [50] also reported that *L. plantarum* strains isolated from wine were capable of degrading cadaverine. At present, however, investigations on cadaverine-degrading activity of *L. brevis* are rarely found in literature.

As for change in spermine content, there appeared difference among groups of *Kkakdugi* and *Chonggak* kimchi. In PC and LB groups of both radish kimchi, a gradual decrease of spermine content was observed over the fermentation period, and the content was relatively lower than that in the other groups of both types of radish kimchi. This implies that *L. brevis* could be able to degrade spermine, although relevant reports are scarce to date. It is worth nothing that in B, C, and LP groups, spermine content decreased for day 1 of fermentation and slightly increased thereafter in *Kkakdugi*, whereas that in *Chonggak* kimchi increased for day 1 and slightly decreased thereafter. The different kinetics of spermine formation seems to result from the complex combinations of factors mentioned above. Therefore, it would be interesting in a future study to identify the factors (and combinations thereof) associated with BA formation or degradation by LAB strains during fermentation of *Kkakdugi* and *Chonggak* kimchi. The factors may involve time-related successional changes and/or interactions of microorganisms during fermentation as well as ingredients of foods and metabolic activities of strains [51]. In addition, recent studies suggested that results of in vitro BA production by food fermenting microorganisms were in disagreement with those of BA formation during fermentation of the corresponding foods [52,53]. In the present study, however, *L. brevis* was considered to be responsible for tyramine formation not only in vitro but also during practical fermentation of *Kkakdugi* and *Chonggak* kimchi.

## 4. Conclusions

The present study indicated that the amounts of BA in most samples of *Kkakdugi* and *Chonggak* kimchi were considered safe for consumption, but some samples contained histamine and total BA at concentrations over toxicity limits (≥100 mg/kg and ≥1000 mg/kg, respectively). It was also found that, while *Myeolchi-aekjeot* seems to be an important source of histamine in both types of radish kimchi, *L. brevis* strains isolated from *Kkakdugi* and *Chonggak* kimchi are highly capable of producing tyramine in assay media. On the other hand, the physicochemical and microbial properties of both radish kimchi revealed weak correlations with BA content in the respective kimchi types in the present study. Through the practical fermentation of *Kkakdugi* and *Chonggak* kimchi, it turned out that *L. brevis* is responsible for tyramine formation, and *Myeolchi-aekjeot* influences histamine and cadaverine content in both radish kimchi. Consequently, this study suggests strategies for reducing BA in radish kimchi: the alteration of the ratio of ingredients used for kimchi preparation, particularly reducing ratio of *Myeolchi-aekjeot* to others, and use of starter cultures other than tyramine-producing *L. brevis* strains, especially BA-degrading LAB starter cultures. Studies on other contributing factors influencing the intensity of BA production by LAB are also required to understand complex kinetics of BA formation in the kimchi.

## Figures and Tables

**Table 1:** Ingredients used for preparation of *Kkakdugi* and *Chonggak* kimchi.

Ingredients (g)	Salted Radish	Red Pepper Powder	Garlic	Ginger	Sesame Seed	Sugar	Glutinous Rice Paste	*Myeolchi-aekjeot*	*Saeu*-*jeotgal*
*Kkakdugi*	100	3	3	1.5	1	2	5	2	2
*Chonggak* kimchi	100	3.5	3	1.5	0.5	1.5	4	2	2

**Table 2:** BA content in two types of radish kimchi samples: *Kkakdugi* and *Chonggak* kimchi.

Samples ^2^	BA Content (mg/kg) ^1^							
	Trp	Phe	Put	Cad	His	Tyr	Spd	Spm
KD1	ND ^3^	ND	10.85 ± 1.17 ^4^	2.57 ± 0.62	18.75 ± 1.16	2.97 ± 0.33	12.27 ± 0.98	0.56 ± 0.96
KD2	ND	1.93 ± 1.69	563.59 ± 45.64	ND	127.78 ± 26.78	14.73 ± 1.96	12.66 ± 2.75	ND
KD3	ND	ND	19.00 ± 2.00	6.10 ± 0.40	24.50 ± 4.00	10.80 ± 0.40	ND	ND
KD4	ND	0.86 ± 1.49	97.45 ± 77.05	3.15 ± 5.46	40.82 ± 29.05	21.67 ± 17.81	5.30 ± 4.85	3.10 ± 2.82
KD5	ND	15.24 ± 1.87	982.32 ± 19.42	124.60 ± 108.78	67.84 ± 17.46	76.95 ± 4.25	16.76 ± 0.87	1.48 ± 0.08
Average	ND	3.61 ± 6.55	334.64 ± 427.97	27.28 ± 54.44	55.94 ± 44.45	25.42 ± 29.59	9.40 ± 6.68	1.03 ± 1.31
CG1	ND	ND	8.97 ± 2.02	2.38 ± 2.12	38.61 ± 6.03	4.85 ± 4.60	9.22 ± 2.16	20.74 ± 3.47
CG2	ND	ND	3.89 ± 1.68	2.00 ± 0.77	8.24 ± 2.09	0.79 ± 0.69	8.27 ± 2.90	2.12 ± 0.53
CG3	12.30 ± 6.30	ND	175.10 ± 7.30	55.40 ± 2.80	46.30 ± 6.70	18.70 ± 2.40	7.70 ± 5.50	ND
CG4	9.10 ± 7.10	1.10 ± 1.00	303.70 ± 20.20	148.50 ± 9.00	69.30 ± 20.90	11.10 ± 2.20	6.10 ± 3.70	8.30 ± 5.60
CG5	23.70 ± 6.10	2.80 ± 1.20	853.70 ± 36.80	112.10 ± 3.60	131.20 ± 7.90	7.00 ± 2.20	14.00 ± 5.30	ND
Average	9.02 ± 9.86	0.78 ± 1.23	269.07 ± 349.93	64.08 ± 65.51	58.73 ± 46.02	8.49 ± 6.80	9.06 ± 2.99	6.23 ± 8.79

^1^ Trp: tryptamine, Phe: β-phenylethylamine, Put: putrescine, Cad: cadaverine, His: histamine, Tyr: tyramine, Spd: spermidine, Spm: spermine; ^2^ KD: *Kkakdugi* (diced radish kimchi), CG: *Chonggak* kimchi (ponytail radish kimchi); ^3^ ND: not detected (<0.1 mg/kg); ^4^ mean ± standard deviation.

## Supplementary Materials

The following is available online at http://www.mdpi.com/2304-8158/8/2/73/s1, Figure S1: Scheme of procedure for BA analysis.
