# Supplementary figures and images for: The Occurrence of Biogenic Amines and Determination of Biogenic Amine-Producing Lactic Acid Bacteria in Kkakdugi and Chonggak Kimchi
Source: Foods. 2019 Feb 14;8(2):73. doi: 10.3390/foods8020073 (PMC6406508; doi:10.3390/foods8020073)

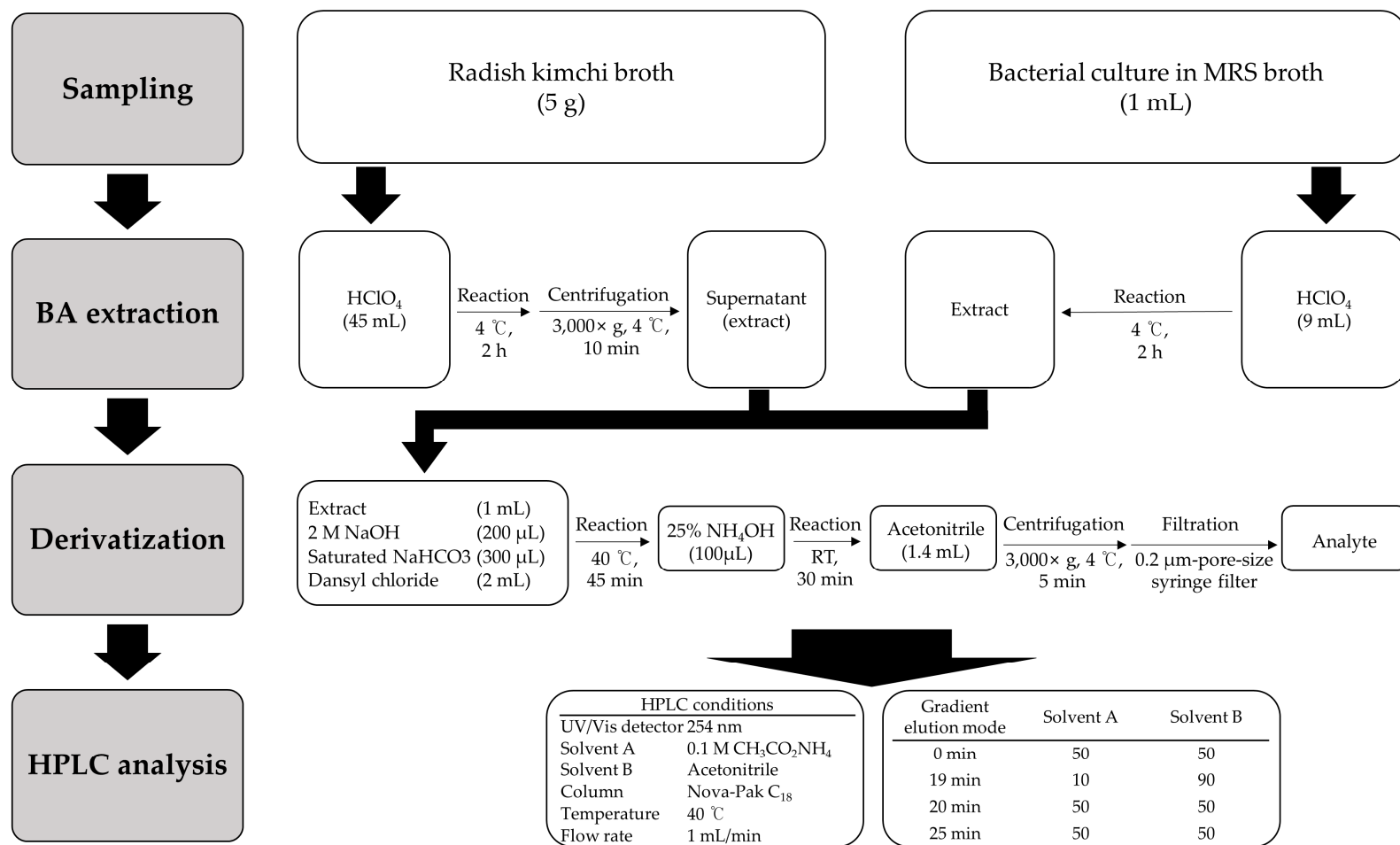

**Figure S1.** Scheme of procedure for BA analysis.

Supplement: Supplementary file 1 [file foods-08-00073-s001.pdf]
